# Draft Genome Sequence of the Dimorphic Fungus *Sporothrix pallida*, a Nonpathogenic Species Belonging to *Sporothrix,* a Genus Containing Agents of Human and Feline Sporotrichosis

**DOI:** 10.1128/genomeA.00184-16

**Published:** 2016-03-31

**Authors:** Enrico D’Alessandro, Domenico Giosa, Lilin Huang, Jing Zhang, Wenchao Gao, Balazs Brankovics, Manoel Marques Evangelista Oliveira, Fabio Scordino, Carla Lo Passo, Giuseppe Criseo, Anne D. van Diepeningen, Huaiqiu Huang, G. Sybren de Hoog, Orazio Romeo

**Affiliations:** a Department of Veterinary Sciences, Division of Animal Production, University of Messina, Messina, Italy; b Department of Chemical, Biological, Pharmaceutical and Environmental Sciences, University of Messina, Messina, Italy; c Department of Dermatology and Venereology, Third Affiliated Hospital of Sun Yat-sen University, Guangzhou, Guangdong, China; d CBS-KNAW Fungal Biodiversity Centre, Utrecht, The Netherlands; e Laboratório de Micologia, Instituto Nacional de Infectologia Evandro Chagas, Fundação Oswaldo Cruz, Rio de Janeiro, Brazil; f IRCCS Centro Neurolesi “Bonino-Pulejo,” Messina, Italy

## Abstract

*Sporothrix pallida* is considered to be a mostly avirulent environmental fungus, phylogenetically closely related to the well-known pathogen *Sporothrix schenckii*. Here, we present the first assembly of its genome, which provides a valuable resource for future comparative genomic studies between nonpathogenic and pathogenic *Sporothrix* spp.

## GENOME ANNOUNCEMENT

In recent years, the fungal genus *Sporothrix* has aroused considerable interest because of the worldwide emergence of some pathogenic species that cause sporotrichosis, a mycosis that affects humans and other mammals (1, 2). The best-known species of the group is *Sporothrix schenckii*, a thermodimorphic fungus with a worldwide distribution, even though the infection that it causes is more common in tropical and subtropical regions (2, 3). However, recent studies (4–6) have shown that the global *S. schenckii* population is a complex of cryptic species comprising four different closely related taxa with diverse degrees of virulence and pathogenicity (7), while occasional opportunists are found elsewhere in the genus.

*Sporothrix pallida* (formerly *Sporothrix albicans*) is considered an avirulent environmental species of the genus *Sporothrix*, and although one case of human keratitis has recently been reported (8), it was found to be nonpathogenic for mice and represents certainly a less versatile pathogen when compared to other members of the *S. schenckii* complex (7).

One of the main difficulties in the study of these fungi is the lack of exhaustive genomic information, which limits our understanding of their basic biology, including their interactions with the mammalian host. Recently, the genome sequences of *S. schenckii* and *S. brasiliensis* have been released (9, 10), and we can now add the *S. pallida* genome in order to provide essential data for future comparative genomic studies among highly pathogenic species and closely related species with reduced or absent virulence.

The genome of *S. pallida* strain SPA8 (5, 11) was sequenced using Ion Torrent (PGM) (318-chip) and Illumina HiSeq 2000 technologies. For sequencing, we constructed four different DNA libraries: one library was generated for single-read sequencing on an Ion PGM machine, while three libraries, with different insert sizes (200 bp, 500 bp, and 6 kb), were used for paired-end Illumina sequencing. Before assembly, raw reads were processed to remove adapters and polyclonal sequences and subsequently filtered and trimmed using FASTX-Toolkit version 0.0.14 (http://hannonlab.cshl.edu/fastx_toolkit) to remove sequences with low Phred-scores (cutoff quality score: ≥20). The final data set used for assembly contained 4,113,066 quality-controlled Ion PGM reads and 21,915,508, 16,647,776, and 16,763,936 clean reads obtained from 200-bp, 500-bp, and 6-kb Illumina libraries, respectively.

The *S. pallida* genome was assembled *de novo* using MIRA version 4.0.2 (12) and SPAdes (13). The assembled genome resulted in 432 contigs (>200 bp; largest contig, 860,569 bp; *N*_50_, 224,849 bp) with a total consensus length of 37,819,765 bp (G+C content: 52.8%) at 50× coverage. A total of 11,356 protein-encoding genes were predicted *ab initio* by AUGUSTUS (14), and 151 putative tRNA genes were found by tRNAscan-SE (15). The entire mitochondrial genome, identified using GRAbB (16), was contained in a single contig (Contig_215) of our assembly and was found to be circular.

Hence, the genome of the nonpathogenic *S. pallida* genome proves to be approximately 5 Mb larger than the genomes of its human pathogenic relatives (9, 10). Further genomic analysis will be of great help to understand the genetics and evolution of pathogenic and nonpathogenic *Sporothrix* spp.

### Nucleotide sequence accession numbers.

This whole-genome shotgun project has been deposited at DDBJ/EMBL/GenBank under the accession number JNEX00000000. The version described in this paper is the second version, JNEX02000000.
